# Fabrication and Characterization of Thin Metal Films Deposited by Electroless Plating with Organic Additives for Electrical Circuits Applications

**DOI:** 10.3390/mi14061151

**Published:** 2023-05-29

**Authors:** Nikita S. Buylov, Nadezhda V. Sotskaya, Oleg A. Kozaderov, Khidmet S. Shikhaliev, Andrey Yu. Potapov, Vladimir A. Polikarchuk, Sergey V. Rodivilov, Vitaly V. Pobedinskiy, Margaryta V. Grechkina, Pavel V. Seredin

**Affiliations:** 1 Laboratory of Organic Additives for the Processes of Chemical and Electrochemical Deposition of Metals and Alloys Used in the Electronics Industry, Voronezh State University, University sq. 1, Voronezh 394018, Russia; buylov@phys.vsu.ru (N.S.B.); nvs-vrn@mail.ru (N.V.S.); kozaderov@vsu.ru (O.A.K.); shikh1961@yandex.ru (K.S.S.); pistones@mail.ru (A.Y.P.); polikarchyk@mail.ru (V.A.P.); 2 Department of Solid-State Physics and Nanostructures, Voronezh State University, University sq. 1, Voronezh 394018, Russia; 3 Research Institute of Electronic Technology, St. Staryh Bolshevikov, 5, Voronezh 394033, Russia; rsv36@yandex.ru (S.V.R.); pobedinsky@niiet.ru (V.V.P.); 4 Department of Semiconductor and Microelectronics Physics, Voronezh State University, University sq. 1, Voronezh 394018, Russia; grechkina_m@mail.ru

**Keywords:** Ni, P-coating, electrolyte, electroless plating, organic additives, electrical circuits applications

## Abstract

In our work, we studied thin nickel films deposited by electroless plating for use as a barrier and seed layer in the through-silicon vias (TSV) technology. El-Ni coatings were deposited on a copper substrate from the original electrolyte and with the use of various concentrations of organic additives in the composition of the electrolyte. The surface morphology, crystal state, and phase composition of the deposited coatings were studied by SEM, AFM, and XRD methods. The El-Ni coating deposited without the use of an organic additive has an irregular topography with rare phenocrysts of globular formations of hemispherical shape and a root mean square roughness value of 13.62 nm. The phosphorus concentration in the coating is 9.78 wt.%. According to the results of the X-ray diffraction studies of El-Ni, the coating deposited without the use of an organic additive has a nanocrystalline structure with an average nickel crystallite size of 2.76 nm. The influence of the organic additive is seen in the smoothening of the samples surface. The root mean square roughness values of the El-Ni sample coatings vary within 2.09–2.70 nm. According to microanalysis data the phosphorus concentration in the developed coatings is ~4.7–6.2 wt.%. The study of the crystalline state of the deposited coatings by X-ray diffraction made it possible to detect two arrays of nanocrystallites in their structure, with average sizes of 4.8–10.3 nm and 1.3–2.6 nm.

## 1. Introduction

The current pace of technological development is directly dependent on scientific and technological advances in the design of new materials or approaches to manufacturing microelectronic products. Continuously increasing the density of transistors on a chip has enabled the higher performance and reliability of integrated circuits (ICs). Today, the most studied and the cheapest silicon technology for IC fabrication has almost reached the limits of dimensional technology [1]; however, the miniaturization trend for microelectronics products is still reserved. Three-dimensional integration is seen as one of the most promising trends in silicon technology [2,3]. This technology will significantly reduce not only the size of semiconductor devices, but also enhance their energy efficiency and operational speed by reducing the length of interconnections [4,5,6]. One of the key elements in 3D integration technology is the interposer, which is a silicon wafer with through-silicon vias (TSV). The object of TSV technology is to provide vertical electrical connections between different layers of semiconductor devices in a compact package [3]. Along with the high interest in stacked IC assemblies [7,8,9], the 3D integration of microelectromechanical systems (MEMS) and ICs has remained of some interest, allowing for the development of hetero-integrated microsystems with high performance, small size, low cost and multiple functions [10,11].

The most attractive material for filling wells in TSV technology is copper, due to its electrical characteristics. However, the use of copper also has a number of disadvantages in the form of a large difference in thermal expansion coefficient compared with silicon [12] and a high Cu diffusion coefficient, which can lead to the significant deterioration of the electrical characteristics of the devices [13]. To eliminate copper diffusion into silicon, a barrier layer of TiN, TiW, TaN, CoP, CoNiP or NiP is applied to the TSV walls [14,15,16,17]. Copper deposition in TSVs is undertaken by electroplating, which entails the deposition of a seed layer [18]. Usually, barrier and seed layers are deposited by sputtering; however, as the aspect ratio (AR) of TSV increases, this method does not achieve the desired results in the conformal coatings. As a result, the thickness of the barrier layer at the top of the TSV can be significantly thicker than at the bottom of the well. This inhomogeneity can lead to an increase in crystallite size in the barrier layer and consequently worsen its barrier properties for copper diffusion. At the same time, areas not covered by the barrier layer can be formed at the bottom of the well and this also leads to diffusion of Cu into Si. The inhomogeneity in the thickness of the barrier and vacuum layers with thickening in the upper part of the TSV can significantly affect the quality when filling the wells and may lead to the formation of voids at the bottom of the TSV [19]. The CVD method of vapor deposition is well suited to eliminate high AR inhomogeneity in the thickness of barrier and seed layers [20,21], but the application of this method is very costly. Excellent results in terms of simplicity and low cost have been shown by chemical Ni deposition as barrier and seed layer in TSV with high AR [19,22,23,24,25].

El-Ni coatings are alloys of the Ni-P system, and their composition and properties depend on the composition of the electrolyte, which in addition to the nickel salt and hypophosphite ions includes various complexing, buffering and stabilizing additives [26,27]. The need for the latter is due to the possibility of an autocatalytic precipitation reaction not only on the target surface, but also in the volume of electrolyte, which can lead to electrolyte decomposition. Sulphur- and nitrogen-containing organic compounds are most often chosen as stabilizing additives [27,28].

Due to the fact that copper diffusion mainly occurs along barrier layer defects, in particular along grain boundaries, an amorphous nickel coating is a desirable result [19]. Chemical nickel layers with appropriate characteristics are deposited from high quality electrolytes using organic additives. The use of organic additives in the nickel bath is essential to obtain coatings with improved structural, mechanical and morphological properties [26].

In [29] the negative effect of hydrogen on the quality of nickel columns formed for MEMS and CMOS integration is discussed. The additive, while inhibiting the bulk reaction, should not significantly affect the surface reaction. Given that the nature of both volume and surface reactions is the same, selecting effective additives is a complex task and for a particular electrolyte requires certain knowledge of their mechanism of action.

In our research work we synthesized a new organic additive, developed an electrolyte composition and selected the modes of chemical nickel deposition. Using the developed electrolyte, a series of nickel coating samples were obtained and their characteristics for use as a barrier and seed layer in TSV technology were investigated.

## 2. Materials and Methods

The Ni, P-coating chemical precipitation bath contained 0.08 mol/L of nickel chloride, 0.28 mol/L of sodium hypophosphite as reducing agent, 0.2 mol/L amino acetic acid as complexing agent and buffer additive and 0.12 mol/L sodium acetate as a buffer additive. Disodium salt of 4,4dithiobenzene disulfonic acid (DBDA) was used as a stabilizing additive. The coatings were deposited on M1 copper plates. The temperature was 80 °C and the pH was 5.5. A 10% sodium hydroxide solution was used to adjust the pH. Coating deposition rate (V, μm/h) was determined by the gravimetric method and by measuring the weight gain of the samples. The stability coefficient of the electrolyte (k, %) was determined based on an electrolyte composition analysis as a share of the mass of nickel in the coating in relation to the total mass of nickel separated from a solution that included sediment formed in a bath. Adjustment of the chemical nickel deposition solution was carried out every 30 min of operation using the chemical analysis data and by replenishing the concentration of the consumed components to the original level. El-Ni sample designations and electrolyte characteristics are presented in Table 1.

The DBDA additive concentration was determined in consideration of the way that it affects the coating deposition rate, the reaction rate in the electrolyte volume and the coating properties. First of all, the concentration interval at which the additive will not reduce the coating deposition rate was determined. The initial concentration values were set arbitrarily. Then, the reaction rate in the electrolyte volume (electrolyte stability) in the chosen concentration interval was evaluated and the properties of the coatings were studied.

The surface morphology of the obtained nickel coatings was investigated using a JSM6510LV JEOL scanning electron microscope and Solver P47 scanning probe microscope. The elemental compositions of the nickel coatings deposited at different concentrations of organic additive were studied by X-ray microanalysis using an INCA Energy 250 electron microscope attachment.

X-ray diffraction analysis of the El-Ni coatings was carried out using a DRON-4.07 diffractometer with CuKα radiation (λ = 1.54051 Ȧ), samples were scanned at room temperature in the Bragg–Brentano geometry with a step of 0.05° 2θ in the accumulation mode at a point for 2 s. The crystallite size of the nickel coatings was calculated using the Scherrer formula.

## 3. Results and Discussion

### 3.1. Rate of Coating Deposition

The preferred use of El-Ni coatings as barrier and seed layers in TSV technology implies the deposition of the layers with an amorphous structure [30]. In this case, the barrier/seed layer must have high a stability and good adhesion to both Cu and SiO_2_ [31]. Nickel has long proven to be a good material for microelectronics applications, as evidenced by the large number of articles on the subject [32,33,34].

Our task was to obtain El-Ni coatings at different concentrations of DBDA additive in the electrolyte, to study its influence on the deposition rate and phase composition of the coatings, and also surface morphology to evaluate their application as a barrier/seed layer for Cu-TSV production.

The main requirements for selection of the stabilizer additives in the electroless nickel electrolyte include the ability to maintain a high level of electrolyte stability and surface reactivity. By adsorbing to the surface of the resulting deposit, they can affect the rate of phosphorus and nickel deposition reactions, which can in turn change the composition and structure of the coating and its morphology.

**Figure 1:**
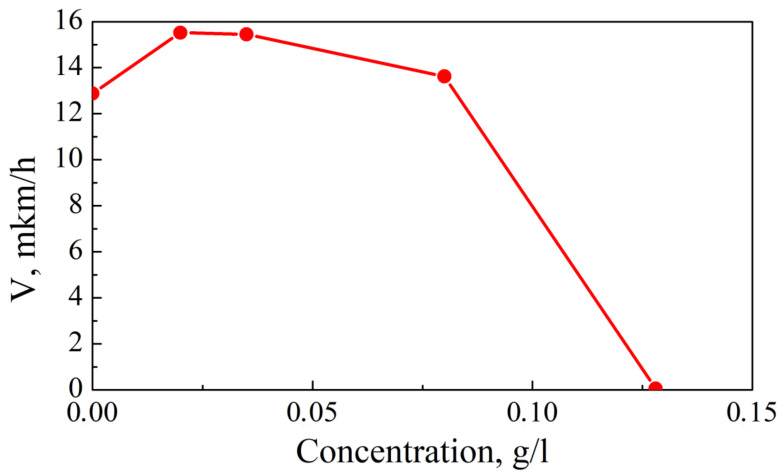
Dependence of coating deposition rate on DBDA concentration.

Figure 1 shows the dependence of the coating deposition rate on the concentration of stabilizing additive. Two areas can be distinguished on the curve. In the region where DBDA concentration is up to 0.08 g/L, the deposition rate is higher than in the original electrolyte, which indicates an increase of catalytic activity of the surface. The maximum deposition rate is observed at 0.02 g/L, at the concentration greater than 0.08 g/L, where the reaction rate decreases sharply, almost to zero, i.e., to the complete cessation.

Ref. [27] indicates that the limiting reaction of the chemical deposition of Ni P-alloys is the oxidation of hypophosphite, which proceeds by the mechanism of dissociative chemisorption with the breaking of the P-H bond. The introduction of the DBDA additive into the solution leads to its adsorption on the surface of the substrate. At low concentrations, this can lead to an increase in the catalytic activity of the surface, since the additive, by polarizing the P-H bond in the hypophosphite ion, accelerates the limiting reaction. With increasing concentration, the additive will displace hypophosphite ions from the surface, which helps to reduce the deposition process up to complete inhibition of the process.

A specific feature of the effect of all stabilizing additives is that, in the bulk of the electrolyte, they inhibit the formation of metallic phase nuclei at the initial stage of the reaction—during the induction period—thus preventing the course of a bulk reaction or significantly reducing its speed. The evaluation criterion is the stability coefficient. All the investigated electrolytes with DBDA additive show a stability coefficient higher than 99.4%, which indicates that it can be used as a stabilizer in this electrolyte.

### 3.2. SEM

**Figure 2:**
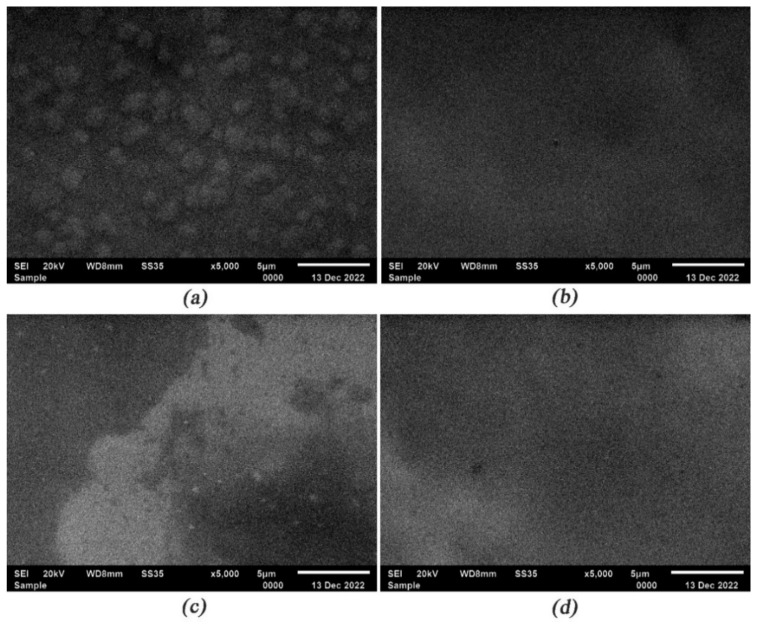
Surface micrographs of El-Ni samples deposited at different concentrations of organic additive. (**a**) DBDA_0, (**b**) DBDA_1, (**c**) DBDA_2, and (**d**) DBDA_3.

The surface of the El-Ni coatings deposited with different concentrations of DBDA in the electrolyte was compared with the surface of the coating deposited under the same conditions from the original electrolyte. Surface microphotographs of El-Ni coatings are shown in Figure 2. The SEM data reveal rare phenocrysts of globular formations of a hemispherical shape with the average size of ~0.9–1.2 µm on a surface of the sample DBDA_0 (Figure 2a) deposited from the electrolyte without the use of an organic additive. The introduction of an organic additive in the amount of 0.02 g/L into the electrolyte leads to a smoothing of the surface of the El-Ni coating of the DBDA_1 sample. A further increase in the content of the additive in the electrolyte with up to 0.08 g/L has no noticeable effect on the surface morphology of the samples DBDA_2 and DBDA_3.

The effect of the organic additive concentration on the phosphorus content of the El-Ni coatings was assessed by microanalysis. El-Ni coatings deposited from the original electrolyte contained 9.78 wt.% of phosphorus (Table 2). The introduction of a small concentration of the developed organic additive into the electrolyte reduced the concentration of phosphorus in the deposited coatings to ~6.2 wt.%. Thus, a variation of concentration of an organic additive in the electrolyte from 0.02 g/L to 0.035 g/L does not lead to the changes of morphology and the phosphorus content in the deposited coatings. Phosphorus content in DBDA_3 coating obtained at the maximum concentration of “DBDA” of 0.08 g/L was reduced to ~4.7 wt.% (Table 2). Earlier in [35,36,37,38] El-Ni coatings were divided into three categories depending on their phosphorus content: low-phosphorus—C_P_ < 5 wt.%, medium-phosphorus—C_P_—5–8 wt.% and high-phosphorus—C_P_ > 9 wt.%. According to the data presented in the mentioned works low-phosphorus coatings contain nickel in micro- or nanocrystalline states (β-phase Ni), medium-phosphorus coatings contain a mixture of β-phase Ni and amorphous γ-phase Ni, and high-phosphorus coatings contain nickel in an amorphous state. Based on the presented data, our coatings with P concentration in the range of 4.7–6.2 wt.% belong to medium-phosphorus and should consist of a mixture of nanocrystalline β-phase Ni and amorphous γ-phase Ni [37,38].

### 3.3. AFM

**Figure 3:**
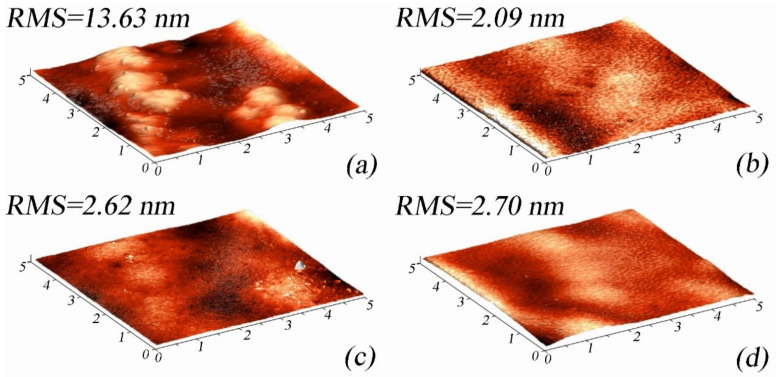
AFM images of 5 × 5 μ El-Ni coating surface areas: (**a**) DBDA_0, (**b**) DBDA_1, (**c**) DBDA_2, and (**d**) DBDA_3.

Figure 3 shows the AFM images of the 5 × 5 μ El-Ni coating surface areas. Analysis of the AFM images shows that the surface of the DBDA_0 sample is characterized by globular formations of hemispherical shape with an average size of about 1 μm, which correlates with the SEM data. In turn, smaller grains with an average size of ~100 nm are detected on the globule surface. The RMS value of the surface roughness of the DBDA_0 sample is 13.63 nm. The addition of DBDA to the electrolyte leads to surface smoothing, as discussed earlier in the discussion of SEM data. However, AFM images of all samples DBDA_1, DBDA_2 and DBDA_3 deposited with organic additive in different concentrations show traces of the original globules but with a blurred topography compared with the sample DBDA_0. The addition of DBDA in the electrolyte also shows the formation of a more developed fine grain structure on the surface of the coatings. The RMS roughness value of the samples DBDA_1, DBDA_2 and DBDA_3 is 2.09 nm, 2.62 nm and 2.70 nm respectively, which is comparable with the results presented earlier [39]. A decrease in the RMS roughness parameter value should lead to improvement in the barrier properties of the deposited coatings [40].

**Figure 4:**
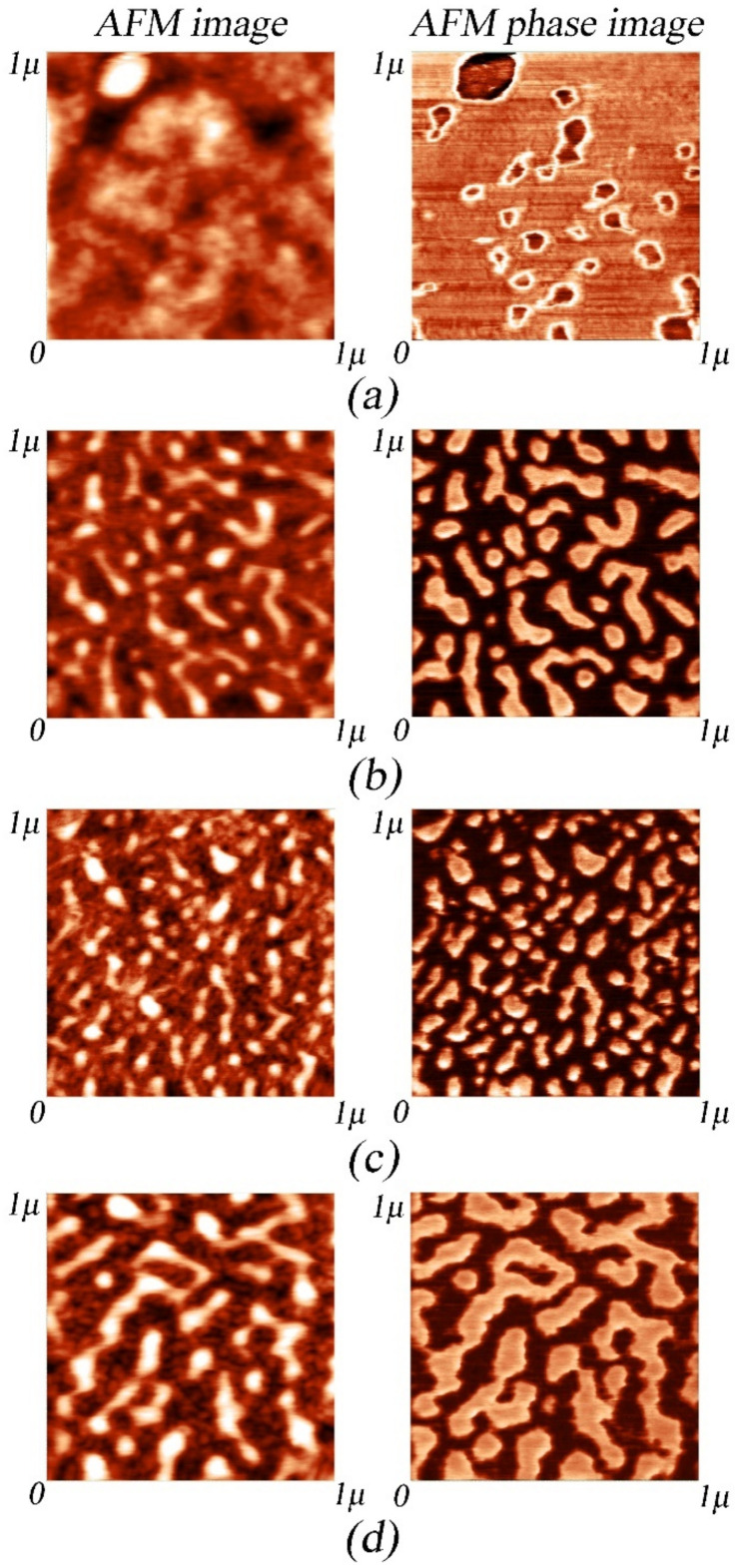
AFM images of topography (left) and phase (right) of 1 × 1 μm El-Ni coating surface areas: (**a**) DBDA_0, (**b**) DBDA_1, (**c**) DBDA_2, and (**d**) DBDA_3.

Figure 4 shows the topography and phase of the 1 × 1 μm El-Ni coating surface areas. A comparison of the topographic and phase AFM data reveals the heterogeneous surface composition of the DBDA_0 sample (Figure 4a), which is characteristic of the amorphous state. At the same time the grains of a rather homogeneous composition, with an average size of 100–300 nm located on the tops of the relief, are present on the surface of the sample. After adding DBDA organic additive to the electrolyte, a fine-grained structure is formed on the surface of the DBDA_1 and DBDA_2 specimens (Figure 4b,c) with an average grain size of 50–70 nm; these tend to form conglomerates of 3–4 grains. At the same time, a clear separation of the grain phases and the surrounding matrix is observed. Increasing the concentration of DBDA in the electrolyte to a maximum value of 0.08 g/L leads to an increase in the grain size up to a value of 70–100 nm (Figure 4d). Along with the large grains on the surface of the DBDA_3 sample, formation of the smaller grains with an average size of ~20 nm is observed.

### 3.4. XRD

**Figure 5:**
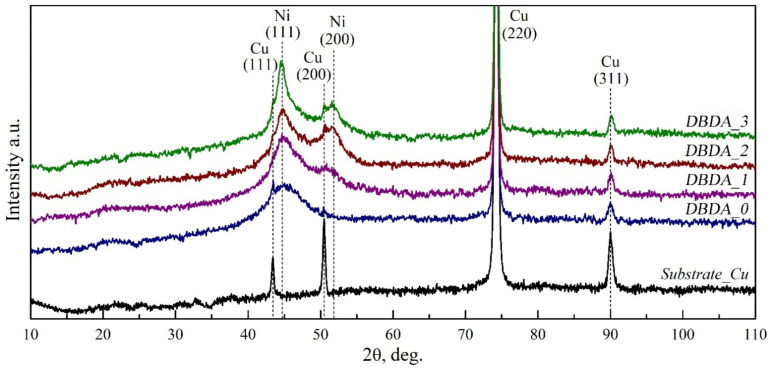
X-ray diffractograms of the substrate (Substrate_Cu) and El-Ni samples deposited at different concentrations of organic additive (DBDA_0, DBDA_1, DBDA_2, DBDA_3).

Experimental diffractograms of copper substrate and El-Ni coatings deposited from the electrolyte containing the developed organic additive in different concentrations (Table 1) are shown in Figure 5. The reflections observed at the angles 2θ = 43.36, 50.48, 74.19, 89.98 correspond to those from Cu (111), (200), (220), (311) substrate planes respectively (ICDD 00-004-0836). Broad maxima in the region of angles 2θ = 44.66, 51.83 belong to the investigated nickel coating and correspond to reflections from planes (111) and (200) of the Ni with a face-centered cubic (hcc) lattice (ICDD 00-004-0850). 

The deposition of El-Ni coatings is always accompanied by the formation of Ni-P bonds [27,28]. According to the literature data [41,42,43], the concentration of phosphorus in the El-Ni coating significantly affects the shape of the Ni X-ray diffraction maximum. With an increase in the P concentration in the El-Ni coating, the size of the Ni crystallites decreases, which leads to a broadening of the Ni X-ray diffraction maximum. Thus, we conclude that the experimental results obtained by X-ray diffraction (Figure 5) indicate the nanocrystalline state of the studied El-Ni coatings.

With an increasing concentration of organic additive “DBDA” in the electrolyte the degree of crystallinity of El-Ni coatings increases. This is manifested in the structuring of the profile of the diffraction line Ni (111) and the increased intensity of reflection from the Ni (200) plane. As can be seen from Figure 5, at the angle value of 2θ = 44.66 in the DBDA_0 sample obtained without the “DBDA” additive, a broad reflection corresponding to Ni (111) is observed. Further, with increasing content of the additive, a narrowing of and an increase in the intensity of the said reflection is observed, and attains a maximum resolution at the additive content of 0.08 g/L. This effect indicates the more ordered structure of the nickel coatings obtained at the maximum organic additive content.

It has been reported in [35,41,44,45] that phosphorus has a low solubility in nickel and hence the Ni lattice is distorted when nickel and phosphorus are co-deposited. In this case, P tends to accumulate at the grain boundaries, suppressing their growth and resulting in a broadening of the diffraction lines. Increasing the concentration of our developed organic additive in the chemical nickel electrolyte leads to a decrease of phosphorus content in the Ni-P coating and is reflected in the X-ray diffraction results accordingly.

The reflections of the nickel phosphide phase (Ni_3_P) are located near those of the hcc Ni and Cu (111), (200) substrates [42,46]. It is not possible to unambiguously determine the presence of the Ni-P phase from the overview diffractograms, therefore the 30–60° 2θ range will be considered in detail.

**Figure 6:**
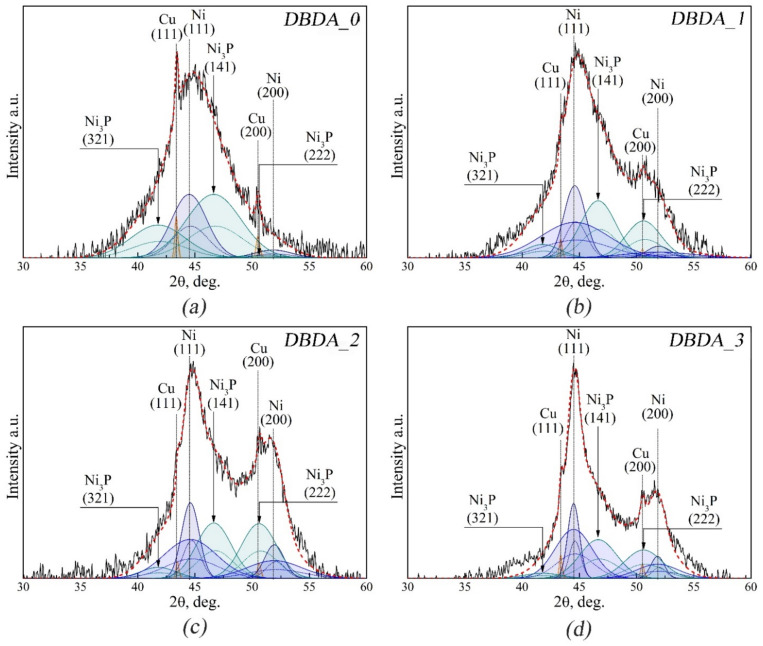
Simulation of X-ray diffraction lines of El-Ni samples deposited at different concentrations of organic additive ((**a**) DBDA_0, (**b**) DBDA_1, (**c**) DBDA_2, (**d**) DBDA_3) in the angle range of 2θ = 30–60°.

Figure 6 shows diffractograms of the deposited El-Ni coatings in the range of 2θ angles 30–60°. The diffractometer “DRON 4.07” involved in these investigations is not equipped with a monochromator crystal, therefore, the contribution of Kα1 and Kα2 components was considered when modeling. The solid lines in Figure 6 show the Kα1 component, the dotted lines show Kα2. Based on the results of simulations of the indicated X-ray diffractogram areas, alongside those of the substrates of Cu (111), (200) and of Ni (111), (200), all lines with nickel coatings, regardless of the presence or absence of our developed organic additive, were found to contain nickel phosphide (Ni3 P) (ICDD 00-074-3245). The simulations were performed considering the contribution of Cu (111), (200) lines with fixed half-widths of FWHM Cu (111) = 0.3 and FWHM Cu (200) = 0.36; these values were obtained from simulations of separately obtained substrate lines using the same instrument. The influence of the organic additive “DBDA” is also seen in the orientation of the nickel coating nanocrystallites towards Ni (111) and Ni (200) with a predominant orientation of Ni (111).

Based on the simulation results, using the Scherrer formula (shape factor of the average crystallite—0.94), the average nickel crystallite size in the El-Ni coating was calculated by evaluating the broadening of the Ni (111) diffraction line compared with the same line of the Ni reference sample. The calculated results are presented in Table 3.

As can be seen from the results presented in Table 3, El-Ni coatings with phosphorus concentration C_p_ = 9.78 wt.% deposited from the electrolyte without the addition of an organic additive contain one array of crystallites with a characteristic size of 2.7 nm (sample DBDA_0). Addition of the organic additive to the parent electrolyte leads to the formation of two arrays of nickel crystallites in the El-Ni structure of the coatings with characteristic sizes of 1.3 nm and 4.8 nm (samples DBDA_1 and DBDA_2, respectively) at C_р_ ~6.2 wt.%. When concentration of the organic additive attains its maximum of 0.08 g/l in the electrolyte the size of the crystallites of nickel in the coating appreciably increases and makes 10.3 nm for a coarse array and 2.6 nm for a fine array at C_p_ = 4.71 wt.%.

Our results for the size of large nanocrystallites, in accordance with the phosphorus content in DBDA_1 and DBDA_2, samples correlate well with the data presented in [42,47]. However, along with the large nanocrystallites of 4.8 nm, we also found smaller grains of 1.3 nm, which correspond to the content of β- and γ-phase Ni in our coatings [37,38].

## 4. Conclusions

The use of chemical deposition for barrier and seed layers in high-aspect-ratio TSV is a good alternative to the costly physical vapor deposition (PVD) process. Nickel is well proven both in terms of the ease with which it produces metallic coatings using chemical deposition and in terms of its performance as a barrier and a seed layer for further filling of the through-silicon vias with copper (Cu-TSV). The preferred use of El-Ni coatings as barrier and seed layers in TSV technology implies the deposition of the layers with an amorphous structure.

In our work the influence of our developed organic additive “DBDA” on the surface morphology, structure and phase composition of the deposited El-Ni coatings was evaluated by SEM, AFM and XRD results. El-Ni coatings were deposited on a copper substrate from the initial electrolyte and using different concentrations of the organic additive. In this work, it was found that the surface of the El-Ni coating that was deposited without an organic additive had an irregular topography with rare phenocrysts of globular formations with a hemispherical shape and an RMS roughness value of 13.62 nm. The concentration of phosphorus in the coating C_p_ = 9.78 wt.%. The results of X-ray studies show that the El-Ni coating DBDA_0 has a nanocrystalline structure with an average size of nickel crystallites d = 2.76 nm. By SEM and AFM methods it was found that the effect of the organic additive “DBDA” is manifested in the smoothing of the surface. RMS roughness values of El-Ni coating samples DBDA_1, DBDA_2 and DBDA_3 decrease to 2.09 nm, 2.62 nm and 2.70 nm, respectively. Based on the microanalysis data, the phosphorus concentrations in the DBDA-applied coatings were evaluated. It was found that the phosphorus concentrations decreased to values ~4.7–6.2 wt.%, which allows us to refer to them as medium phosphorous. Through the data of the comparative X-ray diffraction analysis and the combined use of methods involving the mathematical modeling of diffractograms, it is proved that the coatings DBDA_1, DBDA_2 and DBDA_3 are in a nanocrystalline state and contain two arrays of nanocrystallites with average sizes of d = 1.36 nm and d = 4.82 nm in samples DBDA_1 and DBDA_2 and d = 2.60 nm and d = 10.30 nm in sample DBDA_3. 

The developed coatings using the organic additive “DBDA” have a number of positive properties, including a defect-free surface with low roughness value and their nanocrystalline morphological organization. The positive results we obtained show the feasibility of further research on the deposition of El-Ni coatings on a silicon substrate, followed by a study of the barrier properties of the obtained coatings. Fundamental research in the chosen field should make a significant contribution to the optimization of technological operations performed in the deposition of barrier and bare layers, as well as high-aspect-ratio Cu-TSV filling. The use of electroless nickel deposition will simplify and reduce the cost of manufacturing multi-level IC micro-assemblies and 3D integration of MEMS and CMOS.

## Figures and Tables

**Table 1:** El-Ni sample designations and electrolyte characteristics.

Electrolyte Composition	NiCl_2_ 6H_2_O NaH_2_PO_2_H_2_O NH_2_CH_2_COOH CH_3_COONa 3H_2_O			
Sample Numbers	DBDA_0	DBDA_1	DBDA_2	DBDA_3
Concentration of the Organic Additive DBDA, g/L	0	0.02	0.035	0.08

**Table 2:** Relative concentration of Ni and P in El-Ni coatings as a function of organic additive content.

Sample Numbers	Concentration			
	P		Ni	
	at. %	wt.%	at. %	wt.%
DBDA_0	17.05	9.78	82.95	90.22
DBDA_1	10.98	6.11	89.02	93.89
DBDA_2	11.06	6.16	88.94	93.84
DBDA_3	8.57	4.71	91.43	95.29

**Table 3:** Simulation results and average nickel crystallite size in El-Ni coatings calculated with respect to the Ni (111) diffraction line.

Ni					
hkl	N Образца	FWHM Reference	FWHM Result	2θ deg.	d, nm
111 (Kα1)	DBDA_0	0.32	3.6	44.55	2.76
	DBDA_1	0.32	2.2	44.6	4.82
		0.32	7	44.6	1.36
	DBDA_2	0.32	2.2	44.6	4.82
		0.32	7	44.6	1.36
	DBDA_3	0.32	1.2	44.6	10.30
		0.32	3.8	44.6	2.60

## Data Availability

Data are available on request.

## References

[1] Wong H. On the CMOS Device Downsizing, More Moore, More than Moore, and More-than-Moore for More Moore. Proceedings of the 2021 IEEE 32nd International Conference on Microelectronics (MIEL).

[2] Zhao K., Zhao J., Wei X., Guan X., Deng C., Dai B., Zhu J. (2023). Bottom-Up Cu Filling of High-Aspect-Ratio through-Diamond Vias for 3D Integration in Thermal Management. Micromachines.

[3] Chen X., Chen Z., Xiao L., Hao Y., Wang H., Ding Y., Zhang Z. (2022). Fabrication and Electrical Characterization of High Aspect Ratio Through-Silicon Vias with Polyimide Liner for 3D Integration. Micromachines.

[4] Shenglin M., Yufeng J. (2022). TSV 3D RF Integration: High Resistivity Si Interposer Technology.

[5] Noia B., Chakrabarty K. (2014). Design-for-Test and Test Optimization Techniques for TSV-Based 3D Stacked ICs.

[6] Shen W.-W., Chen K.-N. (2017). Three-Dimensional Integrated Circuit (3D IC) Key Technology: Through-Silicon Via (TSV). Nanoscale Res. Lett..

[7] Lau J.H. Evolution, Challenge, and Outlook of TSV, 3D IC Integration and 3d Silicon Integration. Proceedings of the 2011 International Symposium on Advanced Packaging Materials (APM).

[8] Lau J.H. (2011). Overview and Outlook of Through-silicon via (TSV) and 3D Integrations. Microelectron. Int..

[9] Ko C.-T., Chen K.-N. (2010). Wafer-Level Bonding/Stacking Technology for 3D Integration. Microelectron. Reliab..

[10] Du Y., Wu D., Song Z., Liu M., Yang S., Wang Z. (2016). 3-D Integration of MEMS and CMOS Using Electroless Plated Nickel Through-MEMS-Vias. J. Microelectromech. Syst..

[11] Ramm P., Klumpp A., Weber J., Taklo M.M.V. (2010). 3D System-on-Chip Technologies for More than Moore Systems. Microsyst. Technol..

[12] De Messemaeker J., Pedreira O.V., Philipsen H., Beyne E., De Wolf I., Van Der Donck T., Croes K. Correlation between Cu Microstructure and TSV Cu Pumping. Proceedings of the 2014 IEEE 64th Electronic Components and Technology Conference (ECTC).

[13] Bea J., Lee K., Fukushima T., Tanaka T., Koyanagi M. (2011). Evaluation of Cu Diffusion from Cu Through-Silicon Via (TSV) in Three-Dimensional LSI by Transient Capacitance Measurement. IEEE Electron. Device Lett..

[14] Wang Z. (2015). 3-D Integration and Through-Silicon Vias in MEMS and Microsensors. J. Microelectromech. Syst..

[15] O’Sullivan E.J., Schrott A.G., Paunovic M., Sambucetti C.J., Marino J.R., Bailey P.J., Kaja S., Semkow K.W. (1998). Electrolessly Deposited Diffusion Barriers for Microelectronics. IBM J. Res. Dev..

[16] Zhang Z., Hu X., Jiang X., Li Y. (2019). Influences of Mono-Ni(P) and Dual-Cu/Ni(P) Plating on the Interfacial Microstructure Evolution of Solder Joints. Met. Mat. Trans. A.

[17] Magagnin L., Sirtori V., Seregni S., Origo A., Cavallotti P.L. (2005). Electroless Co–P for Diffusion Barrier in Pb-Free Soldering. Electrochim. Acta.

[18] Murugesan M., Mori K., Bea J.C., Koyanagi M., Fukushima T. (2020). High Aspect Ratio Through-Silicon-via Formation by Using Low-Cost Electroless-Ni as Barrier and Seed Layers for 3D-LSI Integration and Packaging Applications. Jpn. J. Appl. Phys..

[19] Murugesan M., Fukushima T., Mori K., Nakamura A., Lee Y., Motoyoshi M., Bea J.C., Watariguchi S., Koyanagi M. Fully-Filled, Highly-Reliable Fine-Pitch Interposers with TSV Aspect Ratio >10 for Future 3D-LSI/IC Packaging. Proceedings of the 2019 IEEE 69th Electronic Components and Technology Conference (ECTC).

[20] Lee K.-W., Wang H., Bea J.-C., Murugesan M., Sutou Y., Fukushima T., Tanaka T., Koike J., Koyanagi M. (2014). Barrier Properties of CVD Mn Oxide Layer to Cu Diffusion for 3-D TSV. IEEE Electron. Device Lett..

[21] Murugesan M., Bea J.C., Lee K.W., Fukushima T., Tanaka T., Koyanagi M., Sutou Y., Wang H., Koike J. Effect of CVD Mn Oxide Layer as Cu Diffusion Barrier for TSV. Proceedings of the 2013 IEEE International 3D Systems Integration Conference (3DIC).

[22] Lee K.W., Nagai C., Nakamura A., Bea J.C., Murugesan M., Fukushima T., Tanaka T., Koyanagi M. Effects of Electro-Less Ni Layer as Barrier/Seed Layers for High Reliable and Low Cost Cu TSV. Proceedings of the 2014 International 3D Systems Integration Conference (3DIC).

[23] Murugesan M., Fukushima T., Koyanagi M. 500 Nm-Sized Ni-TSVwith Aspect Ratio 20 for Future 3D-LSIs_A Low-Cost Electroless-Ni Plating Approach. Proceedings of the 2019 30th Annual SEMI Advanced Semiconductor Manufacturing Conference (ASMC).

[24] Xiao J., Wang F., Li J., Chen Z. (2023). Comparison of Interfacial Reactions and Isothermal Aging of Cone Ni-P and Flat Ni-P with Sn3.5Ag Solders. Appl. Surf. Sci..

[25] Hu Y., Xiong L., Li M., Hang T. (2022). Covalently Formation of Insulation and Barrier Layers in High Aspect Ratio TSVs. Appl. Surf. Sci..

[26] Mohanty U.S., Tripathy B.C., Singh P., Keshavarz A., Iglauer S. (2019). Roles of Organic and Inorganic Additives on the Surface Quality, Morphology, and Polarization Behavior during Nickel Electrodeposition from Various Baths: A Review. J. Appl. Electrochem..

[27] Delaunois F., Vitry V., Bonin L. (2019). Electroless Nickel Plating: Fundamentals to Applications.

[28] Keping H., Fang J.L. (1997). Stabilization Effect of Electroless Nickel Plating by Thiourea. Met. Finish..

[29] Du Y., Song Z., Zhu H., Wang Z. (2015). Fabrication of Ni Microbumps with Small Feature Size on Au Using Electroless Ni Plating with Noncontact Induction. IEEE Trans. Compon. Packag. Manufact. Technol..

[30] Murugesan M., Mori K., Kojima T., Hashimoto H., Bea J.C., Fukushima T., Koyanagi M. Nano Ni/Cu-TSVs with an Improved Reliability for 3D-IC Integration Application. Proceedings of the 2020 31st Annual SEMI Advanced Semiconductor Manufacturing Conference (ASMC).

[31] Li Z., Tian Y., Teng C., Cao H. (2020). Recent Advances in Barrier Layer of Cu Interconnects. Materials.

[32] Lee B., Jeon H., Jeon S.-J., Kwon K.-W., Lee H.-J. (2012). A Study on the Breakdown Mechanism of an Electroless-Plated Ni(P) Diffusion Barrier for Cu/Sn/Cu 3D Interconnect Bonding Structures. J. Electron. Mater..

[33] Lee B., Jeon H., Gan C.L., Lee H.-J. (2016). Thermal Reliability of a Bilayer of Ni(P)/Cu as a Diffusion Barrier for Cu/Sn/Cu Bonding. Jpn. J. Appl. Phys..

[34] Li Y., Wang Z., Li X., Hu X., Lei M. (2019). Growth Behavior of IMCs Layer of the Sn–35Bi–1Ag on Cu, Ni–P/Cu and Ni–Co–P/Cu Substrates during Aging. J. Mater. Sci. Mater. Electron..

[35] Keong K.G., Sha W. (2002). Crystallisation and Phase Transformation Behaviour of Electroless Nickel-Phosphorus Deposits and Their Engineering Properties. Surf. Eng..

[36] Guo Z., Keong K.G., Sha W. (2003). Crystallisation and Phase Transformation Behaviour of Electroless Nickel Phosphorus Platings during Continuous Heating. J. Alloys. Compd..

[37] Duncan R.N. (1996). The Metallurgical Structure of Electroless Nickel Deposits: Effect on Coating Properties. Plat. Surf. Finish..

[38] Fayyad E.M., Abdullah A.M., Hassan M.K., Mohamed A.M., Jarjoura G., Farhat Z. (2018). Recent Advances in Electroless-Plated Ni-P and Its Composites for Erosion and Corrosion Applications: A Review. Emergent Mater..

[39] Choi K.-K., Kee J., Kwon D.-J., Kim D.-K. (2014). Effect of Hydrogen Plasma on Electroless-Plating Ni–B Films and Its Cu Diffusion Barrier Property. J. Nanosci. Nanotechnol..

[40] Wang B., Li C., Liu Y., Lu X. The CMP Effect of Potassium Molybdate with BTA as Compound Corrosion Inhibitor Used in CMP of the TSV Heterogeneous Microstructure. Proceedings of the 2019 China Semiconductor Technology International Conference (CSTIC).

[41] Salicio-Paz A., Ugarte I., Sort J., Pellicer E., García-Lecina E. (2021). Full Optimization of an Electroless Nickel Solution: Boosting the Performance of Low-Phosphorous Coatings. Materials.

[42] Buchtík M., Krystýnová M., Másilko J., Wasserbauer J. (2019). The Effect of Heat Treatment on Properties of Ni–P Coatings Deposited on a AZ91 Magnesium Alloy. Coatings.

[43] Ahmadkhaniha D., Eriksson F., Zanella C. (2020). Optimizing Heat Treatment for Electroplated NiP and NiP/SiC Coatings. Coatings.

[44] Palaniappa M., Seshadri S.K. (2008). Friction and Wear Behavior of Electroless Ni–P and Ni–W–P Alloy Coatings. Wear.

[45] Shu X., He Z., Wang Y., Yin L. (2020). Mechanical Properties of Ni-Based Coatings Fabricated by Electroless Plating Method. Surf. Eng..

[46] Salicio-Paz A., Grande H., Pellicer E., Sort J., Fornell J., Offoiach R., Lekka M., García-Lecina E. (2019). Monolayered versus Multilayered Electroless NiP Coatings: Impact of the Plating Approach on the Microstructure, Mechanical and Corrosion Properties of the Coatings. Surf. Coat. Technol..

[47] Yan M., Ying H.G., Ma T.Y. (2008). Improved Microhardness and Wear Resistance of the As-Deposited Electroless Ni–P Coating. Surf. Coat. Technol..

